# COVID-19 vaccine boosted immunity against Omicron in chronic myeloid leukemia patients treated with tyrosine kinase inhibitors

**DOI:** 10.1038/s41375-022-01787-8

**Published:** 2022-12-17

**Authors:** Dragana Milojkovic, Catherine J. Reynolds, Diana Mūnoz Sandoval, Franziska P. Pieper, Siyi Liu, Corinna Pade, Joseph M. Gibbons, Áine McKnight, Sandra Loaiza, Renuka Palanicawander, Andrew J. Innes, Simone Claudiani, Jane F. Apperley, Daniel M. Altmann, Rosemary J. Boyton

**Affiliations:** 1 Department of Immunology and Inflammation, Imperial College London, London, UK; 2 Department of Infectious Disease, Imperial College London, London, UK; 3 Blizard Institute, Barts and the London School of Medicine and Dentistry, Queen Mary University of London, London, UK; 4 Imperial College NHS Healthcare Trust, London, UK; 5 Lung Division, Royal Brompton Hospital, Guy’s and St Thomas’ NHS Foundation Trust, London, UK

**Keywords:** Immunology, Chronic myeloid leukaemia

SARS-CoV-2 infection in cancer patients is associated with increased morbidity and mortality, their status defined as ‘clinically extremely vulnerable’ (CEV) with prioritisation for vaccine booster programs, including fifth doses [1]. For patients with Chronic myeloid leukemia (CML) there is uncertainty about their ability to mount a protective immune response against SARS-CoV-2 infection after COVID-19 vaccination. A retrospective study of 8665 CML patients has reported lower COVID-19 mortality than seen in other haematological malignancy [2].

Specific concern over vaccine responsiveness in this group relates to the tyrosine kinase inhibitors (TKI) used in CML therapy being associated with altered B cell immunity. Following influenza or pneumococcal vaccination, TKI-treated CML patients showed impaired antibody (Ab) and memory B cell (MBC) responses, while T cell responses were intact [3]. This was attributed to off-target drug inhibitory effects on B cell signalling kinases. Other cohort studies of cancer patients treated with agents that impair B cell immunity have shown prolonged COVID-19 disease and delayed viral clearance [1, 4]. However, studies measuring spike Ab binding after two vaccine doses [5], and serological and T cell responses in a small number of CML patients (*n* = 16) after the first vaccine dose suggested that the majority respond normally [6]. At a time of continued uncertainty about SARS-CoV-2 infection risk among specific CEV patient groups, detailed immune response analysis following COVID-19 vaccination is critical to inform patients and clinicians when making decisions about shielding and COVID-19 vaccination booster doses.

We undertook a prospective, longitudinal follow-up analysis of T and B cell immunity after COVID-19 vaccination in 62 TKI-treated CML patients and 44 age/sex matched healthy controls. Participants received two doses of either the AstraZeneca (*n* = 22, CML/TKI group) or Pfizer vaccine (*n* = 40, CML/TKI group) followed by a third Pfizer dose (Fig. 1A, S1). TKIs included imatinib, second generation TKIs (nilotinib, dasatinib and bosutinib), ponatinib and asciminib. We measured T cell responses to an ancestral spike peptide pool, S1 receptor-binding domain (RBD) Ab binding, virus neutralizing (nAb) IC50 (ancestral and B.1.1.529 (Omicron BA.1)), and memory B cell (MBC) frequency, as previously described [7–10]. Immune responses were compared at different timepoints after the first, second and third COVID-19 vaccine dose.

**Fig. 1:**
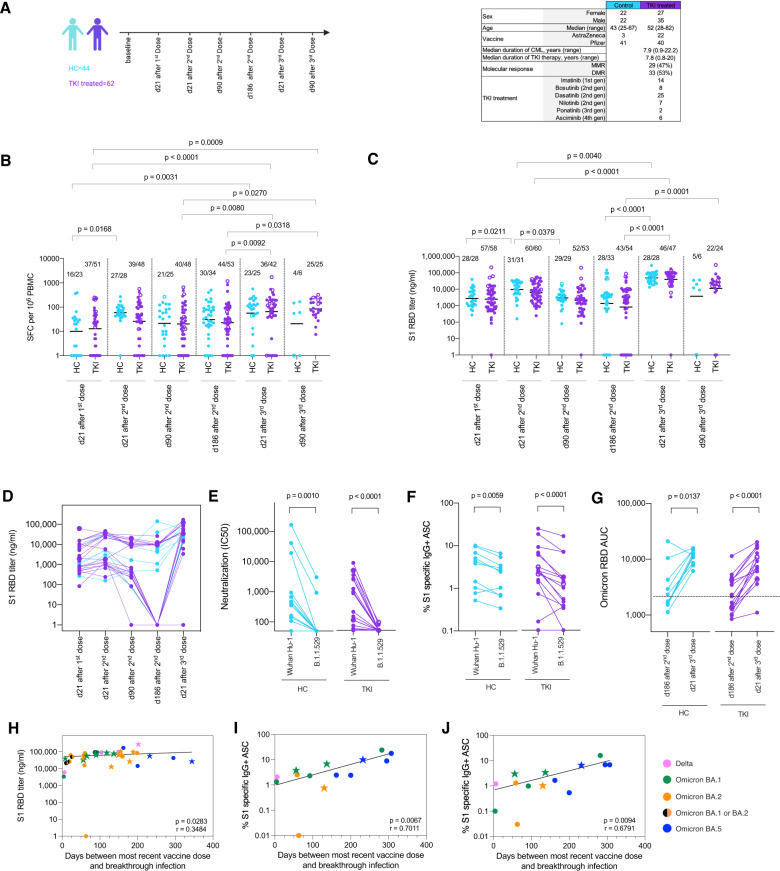
T and B cell responses to SARS-CoV-2 spike antigen after COVID-19 vaccination in CML patients receiving TKI treatment and age/sex matched healthy controls. **A** Graphic summary of the COVAX study cohort follow up at several timepoints after the first, second and third dose of COVID-19 vaccine in this prospective, longitudinal investigation of immunity following COVID-19 vaccination in clinically stable CML patients taking TKI treatment and age/sex matched healthy controls. The table reports demographic data, vaccination history, molecular response and TKI treatments across the groups. **B** T cell response against SARS-CoV-2 ancestral spike mapped epitope peptide (MEP) pool and (**C**) serum Ab titer against ancestral SARS-CoV-2 S1 RBD at different timepoints following first, second and third COVID-19 vaccination dose in healthy control donors (blue, *n* = 6–34) and CML patients taking TKI treatment (purple, *n* = 19–60). T cell responses were measured by IFNγ ELISpot and serum Ab titers were assayed by IgG ELISA. The number of individuals in each group at each timepoint making a positive response to peptide pool or with a positive Ab titer is shown above each plot. Horizontal bars depict geometric mean. Individuals who became infected by SARS-CoV-2 during follow-up are shown as open circles. **D**–**G** Data from study participants in the top (healthy control, blue, *n* = 6 and TKI treated CML patients, purple, *n* = 10) and bottom (healthy control, blue, *n* = 6 and TKI treated CML patients, purple, *n* = 10) quartiles of S1 RBD Ab responses. **D** Longitudinal ancestral S1 RBD Ab titers at timepoints following first, second and third dose of COVID-19 vaccine. **E** Neutralisation IC50 of ancestral and B.1.1.529 (Omicron BA.1) live virus at d186 after the second vaccine dose, lines show paired data for each participant. **F** Memory B cell (MBC) frequency against ancestral S1 and B.1.1.529 (Omicron BA.1) S1 protein at d186 after the second vaccine dose, **G** Ab titers against B.1.1.529 (Omicron BA.1) S1 RBD at d186 after the second vaccine dose and d21 after the third vaccine dose. Pre-vaccination sera were used to set the limit of detection for this assay (AUC = 1980), which is represented as a horizontal dashed line. **H** The relationship between serum S1 RBD Ab titer at d21 after the third vaccine dose and the time interval between the most recent vaccine dose and SARS-CoV-2 breakthrough infection in CML/TKI patients (circles, *n* = 23) and healthy control study participants (stars, *n* = 16) were plotted. Participants infected during the UK Delta (pink), Omicron BA.1 (green), Omicron BA.2 (orange), Omicron BA.1or BA.2 (black/orange) or Omicron BA.5 (blue) waves are plotted as filled shapes. The relationship between the frequency of MBC responding to ancestral (**I**) or B.1.1.529 (Omicron BA.1) spike S1 (**J**) at d186 after the second vaccine dose and the time interval between the most recent vaccine dose and SARS-CoV-2 breakthrough infection in CML/TKI patients (circle, *n* = 10) and healthy controls (star, *n* = 4). Statistics were calculated using Prism 9.0. **B**, **C** Kruskal-Wallis and Dunn’s multiple comparison tests. **E**–**G** Wilcoxon signed rank test. **H**–**J** Spearman’s rank correlation. ASC Ab secreting cells, AUC area under the curve, CML chronic myelogenous leukaemia, d day, HC healthy control, PBMC peripheral blood mononuclear cells, RBD receptor binding domain, S1 subunit 1, SFC spot forming cells, TKI tyrosine kinase inhibitor.

There was no significant difference in T cell response assessed by IFNγ ELISpot against spike antigen between CML/TKI treated patients and controls at any timepoint (Fig. 1B). A third vaccine dose resulted in significantly elevated T cell responses against spike antigen compared to after the first dose in CML/TKI treated patients and controls. There was no significant difference in spike S1 RBD Ab binding between CML/TKI treated patients and controls at any of the timepoints studied (Fig. 1C). There was evidence of antibody waning after the second vaccine dose, but spike S1 RBD Ab titers significantly increased from the second to third vaccine dose. There were no significant differences in T cell or antibody responses according to which TKI was being used, however, the number of patients in each group were small and the study was not powered to test this. We next explored concordance across the different timepoints between S1 RBD Ab binding and T cell spike responses for each participant (Fig. S2A, B). S1 RBD titers were ranked from left to right, lowest to highest. The ranked responses of healthy controls (blue) and CML/TKI patients (purple) were interspersed. The magnitude of S1 RBD Ab binding and spike T cell response were discordant whereby individuals with no Ab response showed a range T cell responses against spike (Fig. S2A, B).

To explore the Ab responses further, we selected control and CML/TKI treated patients in the top and bottom quartile of S1 RBD binding titers (Fig. 1D). Longitudinal analysis of S1 RBD binding showed that all CML/TKI patients within the bottom quartile showed pronounced Ab waning such that all were undetectable by d186 after the second dose (Fig. 1D). Nevertheless, almost all showed complete rescue of vaccine induced S1 RBD Ab binding 21d after the third vaccine dose (Fig. 1D). S1 RBD Ab titers from those patients in the top quartile were more stable and titers remained high throughout follow-up and were further boosted by a third vaccine dose. High Ab binding responders showed potent neutralisation of ancestral live virus, but this did not translate into cross-protective neutralization of B.1.1.529 (Omicron, BA.1), except in the case of 2 healthy control participants (Fig. 1E). Thus, even individuals with high S1 RBD Ab titers following two vaccine doses had poor coverage of B.1.1.529 cross-protective neutralizing epitopes at d186 after the second vaccine dose. This is in line with our previous finding that ancestral S1 RBD-binding is a poor proxy for B.1.1.529 (Omicron, BA.1) live virus neutralization [7]. We next considered the extent of cross-recognition of B.1.1.529 S1 by vaccine-primed MBC (Fig. 1F). MBC frequency against ancestral and B.1.1.529 (Omicron, BA.1) S1 was enumerated at d186 after the second vaccine dose. CML/TKI patients and healthy controls showed equivalent MBC frequencies against ancestral S1 protein. In almost all cases, the MBC frequency against B.1.1.529 (Omicron) S1 was lower, but B.1.1.529 (Omicron) specific MBC were detectable in all but two of the samples tested, including from those individuals who were in the lower quartile of S1 RBD Ab responses. With the exception of one CML/TKI patient, all individuals, including those in the lower quartile of S1 RBD titer at day 186 after the second dose, showed significant enhancement of cross-reactive Ab binding against B.1.1.529 (Omicron) 3 weeks after the third dose (Fig. 1G).

There was no difference in the frequency of CML/TKI patients compared to healthy controls (30/49, 61% vs 23/31, 74%; chi square, 1.428, *p* = 0.2320) self-reporting PCR/lateral flow test positive SARS-CoV-2 breakthrough infection during longitudinal follow up. Of those participants who had breakthrough infections, 10/30 of the CML/TKI patients (Ancestral & BA.2; Ancestral & BA.1/2; Alpha & Delta; Delta & BA.1; Delta & BA.5; BA.1 & BA.2; BA.1 & BA.5; BA.1 & BA.5; BA.1/2 & BA.5; BA.2 & BA.5) and 5/23 of healthy controls (Ancestral & BA.2; Alpha & Delta; Alpha & BA.2; BA.1 & BA.5; BA.1 & BA.5) were subsequently re-infected with different SARS-CoV-2 variants.

Higher S1 RBD Ab binding at 21 days after the third vaccine dose was associated with a longer time interval between the most recent vaccine dose and SARS-CoV-2 breakthrough infection (Fig. 1H) suggesting that enhanced boosting of Ab binding levels following COVID-19 vaccination results in more durable protection against SARS-CoV-2 infection. The same was also true for ancestral S1 and B.1.1.529 (Omicron, BA.1) S1 specific memory B cell frequencies (Fig. 1I, J) where those individuals with the highest memory B cell frequency at 186 days after the second vaccine dose had the longest time interval between most recent vaccine dose and breakthrough SARS-CoV-2 infection.

Our prediction based on observations for other vaccines and known effects of TKI on B cell signalling kinases, was that Ab responses after vaccination would be impaired, with T cell immunity remaining intact. In fact, we observed largely intact Ab and T cell responses, with no significant difference between CML/TKI patients and controls. The majority showed potentially protective anti-spike T cell and Ab binding vaccine induced responses of similar magnitude to those of healthy controls. This suggests that the potent activation of immune pathways by mRNA and adenoviral vectored vaccines is largely able to overcome the B cell activation deficits previously observed with analysis of responses to conventional pneumococcal or influenza vaccines. However, since December 2021 the global pandemic has entered a phase of enhanced population vulnerability (even in the triple-vaccinated) due to vaccine escape, resulting in reduced Ab cross-protection against the prevalent B.1.1.529 (Omicron, BA.1) and related sub-variants, BA.2 and BA.5. It was, therefore, of value to probe whether CML/TKI patients had cross-protective B.1.1.529 (Omicron BA.1) MBC and binding Ab. We found that CML/TKI patients had equivalent cross-recognition of B.1.1.529 (Omicron, BA.1) S1 antigen at the level of MBC frequency to healthy control subjects, and that antibody boosting after a third vaccine dose was equally effective in CML/TKI patients compared to healthy controls. Omicron binding MBC are efficiently reactivated following a third dose of ancestral spike vaccine and correlate with a corresponding increase in nAb titers [11]. Breakthrough SARS-CoV-2 infection in our cohort with the longest time interval between most recent vaccination dose and infection was associated with the highest boost in S1 RBD antibody binding after the third vaccine dose and with the highest frequency of MBC 186 days after the second vaccine dose irrespective of infecting variant.

In conclusion, this representative cohort of clinically stable CML patients on TKI treatment, would not have been exceptionally vulnerable during the initial, pre-VOC phase of the COVID-19 pandemic having made good immune responses after 3 doses of COVID-19 vaccine. However, vaccine escape resulting in reduced, vaccine induced cross-protective repertoires against B.1.1.529 (Omicron BA.1) and its subvariants is a significant factor in breakthrough infection, making vaccine boosting in vulnerable populations such as CML/TKI patients important going forward to ensure adequate protection from severe disease, hospitalisation and death. Our study suggests that CML patients on TKI treatment boost Ab and cellular immunity and that those individuals with the biggest boost in Ab titer and memory B cell frequency will have the most durable protection against future SARS-CoV-2 breakthrough infection with a VOC. This coupled with the high rates of vaccine breakthrough infection make uptake of COVID-19 vaccine bivalent booster dose extremely important.

## Supplementary information

Supplemental Information

## Data Availability

The datasets generated and/or analysed during the current study are available from the corresponding author on reasonable request.
