# Supplementary material for: COVID-19 vaccine boosted immunity against Omicron in chronic myeloid leukemia patients treated with tyrosine kinase inhibitors
Source: Leukemia. 2022 Dec 17;37(1):244–7. doi: 10.1038/s41375-022-01787-8 (PMC9758679; doi:10.1038/s41375-022-01787-8)
Supplement: Supplementary file 1 — Supplemental Information [file 41375_2022_1787_MOESM1_ESM.pdf]

Supplemental Information

COVID-19 vaccine boosted immunity against Omicron in chronic myeloid leukemia patients treated with tyrosine kinase inhibitors

Dragana Milojkovic[1†], Diana Mūnoz Sandoval[2†], Franziska P. Pieper[2], Siyi Liu[2], Catherine J Reynolds[2], Corinna Pade[3], Joseph M. Gibbons[3], Áine McKnight[3], Sandra Loaiza[1], Renuka Palanicawander[1], Andrew J Innes[1], Simone Claudiani[1], Jane F Apperley[1], Daniel M Altmann[1], Rosemary J Boyton[2,4*]

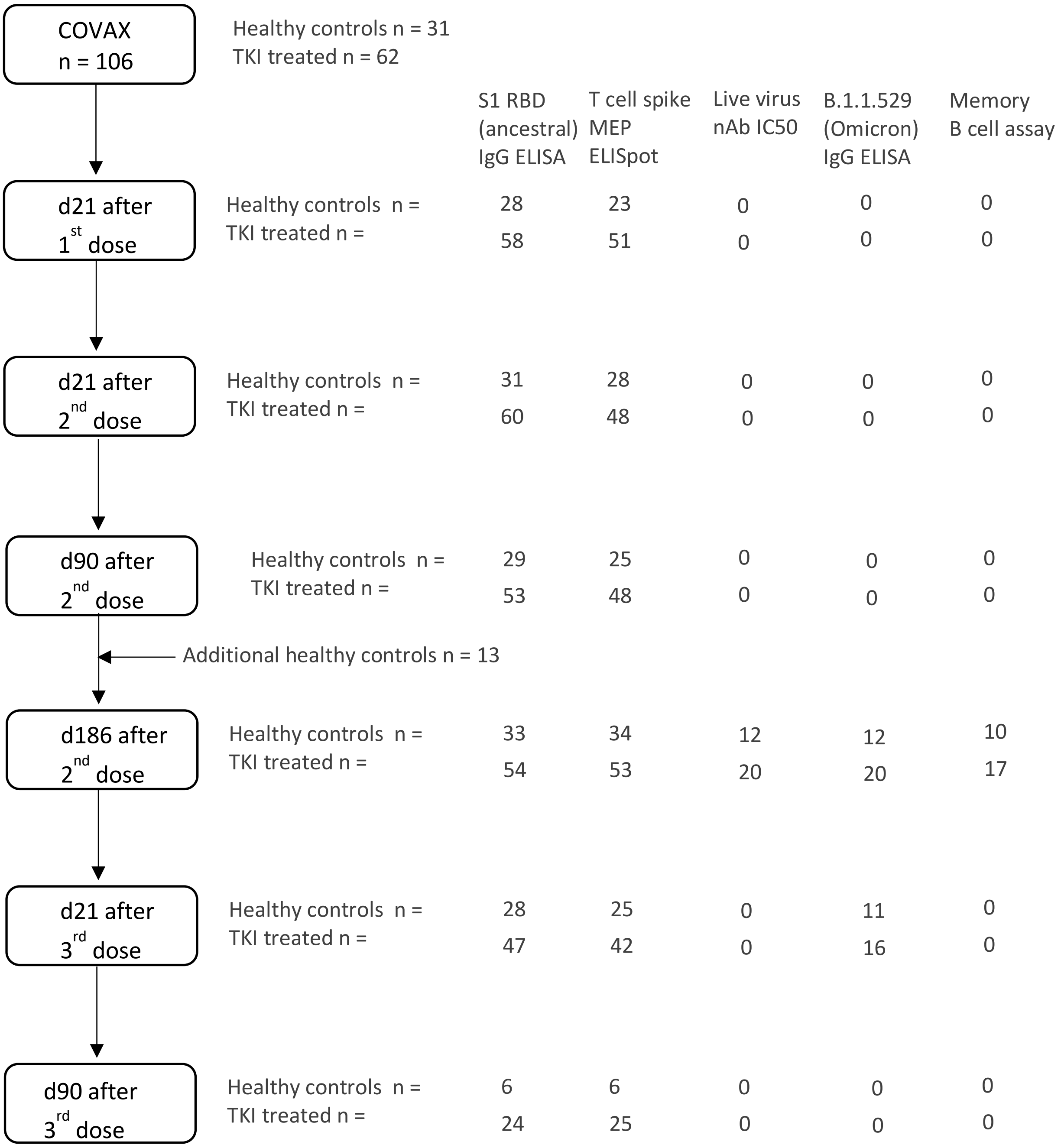

Supplementary Figure S1. **CONSORT diagram of COVAX study.** CONSORT flow diagram showing participant numbers in this prospective, longitudinal study. CML patients taking TKI treatment and age/sex matched healthy controls were recruited.

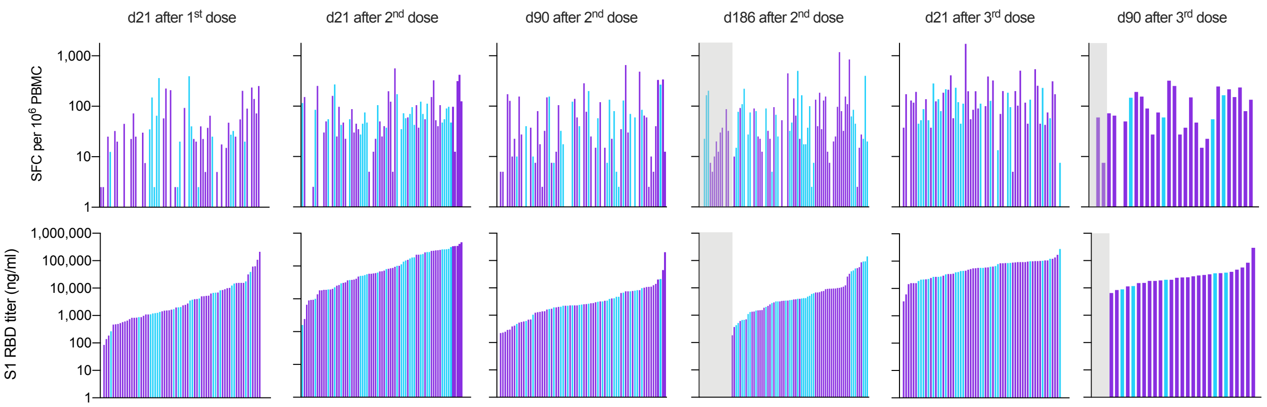

Supplementary Figure S2. T cell and S1 RBD antibody binding responses to SARS-CoV-2 spike antigen are discordant in both CML patients on TKI treatment and age/sex matched healthy controls. **(A)** T cell responses to spike MEP pool and (**B**) serum S1 RBD Ab titers by individual, ranked by increasing magnitude of S1 RBD Ab titer [control donor (blue, n = 6-34) and CML patient taking TKI treatment (purple, n = 19-60)]. Grey areas in the panels indicate individuals with no detectable S1 RBD Ab. CML, chronic myeloid leukaemia; d, day; MEP, mapped epitope peptide; PBMC, peripheral blood mononuclear cells; RBD, receptor binding domain; S1, subunit 1; SFC, spot forming cells; TKI, tyrosine kinase inhibitor.

Supplementary Methods

Study design and subjects

CML-Co-vax study (Chronic Myeloid Leukemia SARS-CoV-2 Vaccination) study is a prospective observational study (IRAS ID: 280527; REC ID: 21/SC/0016) assessing the immunogenicity of COVID-19 vaccination in clinically stable CML patients taking TKI treatment (imatinib, dasatinib, bosutinib, nilotinib, ponatinib, asciminib; (n=62)) compared to healthy controls (n=44). Both groups received two doses of either BNT162b2 or ChAdOx1 nCov-19 (Oxford–AstraZeneca) vaccine 6–12 weeks apart followed by a third dose of BNT162b2 (in accordance with UK government recommendations) (Figure 1A, S1 CONSORT diagram). Inclusion criteria were CML in chronic phase currently taking TKI treatment and in at least cytogenetic remission. Exclusion criteria at the start of the study was a history of laboratory confirmed SARS-CoV-2 infection either by PCR or serological testing or previous vaccination. The median duration of CML and of TKI therapy was 7.9 (range 0.9-22.2) and 7.8 (range 0.8-20.0) years respectively (Figure 1A). Patients and age/sex matched healthy controls were recruited at Hammersmith Hospital from the outpatient clinical service as described previously[5]. T cell responses against spike MEP pool and anti-SARS-CoV-2 spike S1 RBD Ab binding were measured at 21 days after the first, second and third vaccine doses, at 90 and 186 days after the second dose and at 90 days after the third vaccine dose. The 186 day timepoint is the median day pre-3rd vaccine dose. A SARS-CoV-2 infection during the study timeline was defined as a reported positive SARS-CoV-2 PCR or lateral flow test or positive N antibody binding response. Breakthrough infections and re-infections were self-reported and data recorded up to 21st November 2022.

Peptides and recombinant proteins

The spike mapped epitope peptide (MEP) pool is comprised of eighteen 12-20mer peptide epitopes and has been previously described[7-10]. All peptides were synthesised by GL Biochem Shanghai Ltd (China).

Recombinant ancestral Wuhan Hu-1 SARS-CoV-2 S1 protein was purchased from GenScript USA Inc. (product reference: Z03485-1). B.1.1.529 (Omicron) S1 protein (containing the mutations A67V, H69del, V70del, T95I, G142D, V143del, Y144del, Y145del, N211del, L212I, ins214EPE, G339D, S371L, S373P, S375F, K417N, N440K, G446S, S477N, T478K, E484A, Q493R, G496S, Q498R, N501Y, Y505H, T547K, D614G and H655Y) and S1 RBD protein (containing the mutations G339D, S371L, S373P, S375F, K417N, N440K, G446S, S477N, T478K, E484A, Q493R, G496S, Q498R, N501Y and Y505H) were purchased from the Native Antigen Company (product references: REC32006 and REC32007).

IFNγ ELISpot Assays

T cell responses were measured from cryopreserved PBMC samples using a Human IFNγ ELISpot^{PRO} kit (Mabtech 3420-2APT) as previously described[7-10]. Briefly, 200,000 cells per well and cultured for 18-20 hours with either culture media alone, spike MEP peptide pool (10μg/ml/peptide) or anti-CD3 (positive control, Mabtech mAb CD3-2). Assays were run in duplicate. ELISpot plates were developed using a biotinylated anti-human IFNγ detection antibody conjugated to alk-phosphatase (7-B6-1-ALP, Mabtech) and BCIP/NBT-plus phosphatase substrate (Mabtech). Data was collected using an AID classic ELISpot reader (Autoimmun Diagnostika GMBH, Germany). Data analysis was performed in Microsoft Excel. The average of two culture media alone wells was subtracted from spike MEP stimulated wells. Responses lower in magnitude than 2 standard deviations of the control wells value (performed individually for each study participant) was considered a negative response. Results are shown as the difference in spot forming cells per 10^6 PBMC between the negative control and spike MEP stimulation.

Spike S1 RBD ELISA

Serum antibodies against ancestral Wuhan Hu-1 S1 RBD were analysed using a LEGEND MAX™ SARS-CoV-2 Spike RBD Human IgG ELISA Kit (Biolegend, 447707). The assays were performed following manufacturer's instructions. Briefly, serum samples were diluted between 1,000-30,000-fold in assay buffer B and added in duplicate to each pre-coated plate. Serum samples and standards were incubated

for 2 hours at room temperature. Plates were washed 4 times with 1X wash buffer and then incubated with SARS-CoV-2 Spike RBD Human IgG Detection Antibody for 1 hour. Following 4 more washes, plates were incubated for 30 minutes with Avidin-HRP before washing and developing with substrate solution for 10 minutes in the dark. Reactions were terminated with stop solution and absorbance data collected at OD450nm using a Multiskan™ FC Microplate Photometer (Thermo Fisher Scientific). Samples with absorbance values outside the standard curve of each plate were repeated at a higher dilution. The standard curve for each plate was used to define titers in ng/ml for each sample.

B.1.1.529 (Omicron) S1 RBD ELISA

Serum antibodies against B.1.1.529 (Omicron) S1 RBD were measured using Nunc 96-well immune ELISA plates coated with 1ug/ml of B.1.1.529 Spike RBD protein in carbonate buffer (Sigma Aldrich) for 2 hours at 37^{0}C and then blocked with PBS containing 1% bovine serum albumin (BSA) for a further 1 hour. A four-point dilution series (1:10) was run in duplicate for each serum sample, starting at a dilution of 1/100, and plates were incubated overnight at 4^{0}C. ELISA assays were developed using biotinylated mouse anti-human IgG (BD Pharmingen, 555785, 1:1000 dilution), streptavidin horseradish peroxidase (HRP, Biotechne, DY998, 1:200 dilution) and 3,3',5,5-Tetramethylbenzidine (TMB) substrate (Sigma Aldrich, T0400). Plates were washed four times with PBS+0.05% Tween 20 between every incubation step of the protocol. Reactions with substrate were stopped after 10 minutes by the addition of 0.18M H_2SO_4. Absorbance data was collected at OD450nm using a Multiskan™ FC Microplate Photometer (Thermo Fisher Scientific) and data analysis performed in Prism 9.0 for Mac OS (GraphPad). Data for serial dilutions were plotted and area under the curve calculated for each individual serum sample.

Ancestral Wuhan Hu-1 and B.1.1.529 (Omicron) live virus microneutralization assays

All SARS-CoV-2 propagation and microneutralization assays were performed in a containment level 3 facility. Viral stocks of ancestral Wuhan Hu-1 and B.1.1.529 (Omicron) isolates were prepared and titrated and microneutralization assays carried out as previously described[7]. For microneutralization assays we used VeroE6 cells seeded in 96-well plates 24h before infection. Participant sera was heat inactivated

and duplicate titrations of each sample incubated at 37^0C for 1 hour with 3 x 10^4 FFU of ancestral Wuhan Hu-1 or B.1.1.529 (Omicron) SARS-CoV-2 virus (TCID100). Serum/virus preparations were added to pre-seeded VeroE6 cells and incubated at 37^0C for 72h before fixing surviving cells in formaldehyde and staining with 0.1% (wt/vol) crystal violet solution. Absorbance data was collected at OD570nm using a CLARIOStar Plate Reader (BMG Labtech). Pooled pre-pandemic sera (dating from before 2008) and pooled sera from previously analysed neutralization positive SARS-CoV-2 convalescent individuals were used as negative and positive controls respectively. Absorbance values for each well were standardized against technical positive (virus only) and negative (cells only) controls on each plate to determine percentage neutralization values. IC50s were determined from neutralization curves plotted using Prism 9.0.

Memory B cell (MBC) ELISpot assays

Memory B cell ELISpot assays were performed as previously described[7]. We used cryopreserved PBMCs pre-cultured at 37^0C for 5 days with 1 μg/ml of the TLR7/8 agonist R848 and 10 ng/ml of recombinant human IL-2 (Mabtech Human Memory B-cell Stimpack 3660-1). On day 4 of culture, ELISpot PVDF plates (Millipore MSIPS4W10) were pre-coated at 4^0C overnight with PBS alone, purified anti-human IgG MT91/145 (10μg/ml, Mabtech 3850-3-250), ancestral Wuhan Hu-1 S1 protein (10μg/ml) or B.1.1.529 (Omicron) S1 protein (10μg/ml). On day 5, plates were washed 5 times with sterile PBS and blocked at 37^0C for 1 hour with RPMI1640 (GibcoBRL) media supplemented with 10% heat inactivated FCS; 1% 100xpenicillin/streptomycin and 1% 100xL-Glutamine solutions (GibcoBRL). Stimulated PBMC cultures were washed twice in complete media and seeded at 15,000-7,500 cells/well for anti-human IgG coated wells or 150,000-15,000 cells/well for S1 protein coated wells. All assays were run in duplicate and wells coated with PBS alone, anti-human IgG, ancestral Wuhan Hu-1 S1 protein or B.1.1.529 (Omicron) S1 protein. Plates were incubated for 18-20h at 37°C. Assays were developed by successive incubations with 1:200 biotinylated anti-human IgG MT78/145 (Mabtech 3850-6-250) in PBS+0.5% FCS, for 2h at room temperature followed by 1:1000 Streptavidin-ALP (Mabtech 3310-10-1000) in PBS+0.5% FCS for 1h at room temperature and finally BCIP/NBT substrate (Mabtech). Reactions were stopped by washing and drying before analysing on an

AID classic ELISpot plate reader (Autoimmun Diagnostika GMBH, Germany). Plates were washed 5 times with PBS+0.05% Tween 20 (PBST) between steps of the assay development protocol. Data analysis was performed in Microsoft Excel. Spots counted in each well were adjusted according to the number of cells seeded. The number of SARS-CoV-2 S1 antigen specific Ab secreting cells (ASC) was expressed as a percentage of the total number of IgG ASC as calculated using the anti-human IgG control wells.

Statistics and reproducibility

All datasets were assumed to have a non-Gaussian distribution and non-parametric test were used throughout. A Wilcoxon signed rank test was used for paired analysis and unpaired comparisons were done using a Kruskal-Wallis test with Dunn's multiple comparison or a Mann Whitney U test. Correlations were tested using a Pearson's correlation coefficient. Analysis and graphical presentation of the data was performed using GraphPad Prism version 9.0 for Mac OS.
